# Supplementary material for: Metabolic traits predict the effects of warming on phytoplankton competition
Source: Ecol Lett. 2018 Mar 25;21(5):655–64. doi: 10.1111/ele.12932 (PMC6849607; doi:10.1111/ele.12932)
Supplement: Supplementary file 1 [file ELE-21-655-s001.pdf]

Supporting Information for

Metabolic traits predict the effects of warming on phytoplankton competition

Elvire Bestion[1*], Bernardo García-Carreras[2*], Charlotte-Elisa Schaum[1], Samraat Pawar[2**], Gabriel Yvon-Durocher[1**]

Ecology Letters, (2018), doi: 10.1111/ele.12932

[1]Environment and Sustainability Institute, University of Exeter, Penryn, Cornwall TR10 9EZ, UK

[2] Department of Life Sciences, Imperial College London, Silwood Park Campus, Ascot, Berkshire, SL5 7PY, UK

* Joint first authors

** Corresponding authors

Contents

S1. Theory 3

S2. Experimental design 11

Figure S2A. Flow chart of the experimental design 11

Table S2A. Detailed information about the six species. 12

Table S2B. Phosphate concentration levels for each solution, in μmol·L^{-1} and μg·L^{-1}. 12

S3. Discrimination between species in the competition experiment 13

Table S3A. Performance of the discrimination algorithms at day 14. 15

Figure S3A. Example of discrimination between species among pairs of species. 16

Figure S3B. Competition outcomes at day 14. 17

S4. Temperature dependence of the Monod model parameters 18

Table S4A. Metabolic traits for each alga 18

Table S4B: Half-saturation constants (K_S) and degree of nutrient saturation. 18

Table S4C. Results from the GAMs of ln(μmax) as a function of temperature 19

Table S4D. Results from the GAMs of ln(Ks) as a function of temperature 19

S5. Significance of competitive advantage predicted by the model. 20

Figure S5A. Histogram of proportions of competitive advantages correctly predicted for 10,000 random parameter combinations. 20

S6. Robustness of the results to different statistical methods 21

Table S6A. Proportion of competitive advantages correctly predicted by theory using the random forest discrimination method at day 14. 21

Table S6B. Proportion of competitive advantages correctly predicted by theory using the rpart discrimination method at day 14. 22

S7. Quantitative relationship between theoretical and experimental outcomes............................... 23

Figure S7A. Correlation between the observed and predicted competitive advantage at day 14. 23

Table S7A. Results from the linear mixed model investigating observed *R* as a function of predicted *R* at day 14.. 24

Table S7B. Results from the linear mixed model investigating observed *R* as a function of predicted *R* at day 14 excluding pairs involving *Raphidocelis*. 24

Table S7C. Link between observed and predicted *R* at day 14 by species. 24

S8. Nature of species interactions 25

Figure S8A. Distribution of algal communities across an interaction gradient. 26

Table S8A. Deviation from the expected yield per species at day 14. 27

S9. Competitive advantage at day 5 and day 23 28

Table S9A. Proportion of competitive advantages correctly predicted by theory at day 5 using the linear discrimination algorithm. 29

Table S9B. Proportion of competitive advantages correctly predicted by theory at day 23 using the linear discrimination algorithm. 30

S1. Theory

Our objective is to quantify how interspecific differences in metabolic traits affect the competitive advantage of either of a pair of competing phytoplankton species when both species are rare and colonizing (co-invading) a virgin environment. For this, we start with the well-established model of two phytoplankton populations competing for a single limiting nutrient (S) in a chemostat-type environment (Tilman 1977, 1981):

$$\frac{1}{N_a}\frac{\mathrm{d}N_a}{\mathrm{d}t} = (\mu_a - D) = \frac{\mu_{\max,a}\, S}{K_{S,a}+S} - D \qquad (11a)$$

$$\frac{1}{N_b}\frac{\mathrm{d}N_b}{\mathrm{d}t} = (\mu_b - D) = \frac{\mu_{\max,b}\, S}{K_{S,b}+S} - D \qquad (11b)$$

$$\frac{\mathrm{d}S}{\mathrm{d}t} = D(S_0 - S) - \sum_{i=1}^{2} \frac{\alpha_i\, \mu_{\max,i}\, S}{K_{S,i}+S} N_i. \qquad (11c)$$

Here, N_i is the i-th species density (cells·mL^{-1}), μ_i is its realised growth rate (d^{-1}), $\mu_{\max,i}$ is its maximum growth rate in nutrient saturated conditions (d^{-1}), $K_{S,i}$ is the half-saturation constant (µmol·L^{-1}) (the nutrient concentration at which realised growth is $\mu_{\max}/2$; a measure of performance at low nutrient concentrations), S is the nutrient concentration (µmol·L^{-1}), D is dilution rate, and S_0 is the inflow concentration of nutrients. The constant α_i converts units of nutrient to phytoplankton cell units (1000·µmol·cell^{-1}); that is, it is the inverse of the number of phytoplankton cells produced per unit of resource.

The Monod equation's parameters μ_{max} and K_S are functional traits that depend on the species' physiology, and play an important role in shaping competitive dynamics in phytoplankton communities (Tilman 1981; Bulgakov & Levich 1999). Because the nutrients are not replenished in our colonisation experiments, $D = 0$, leaving

$$\frac{1}{N_a}\frac{\mathrm{d}N_a}{\mathrm{d}t} = \mu_a = \frac{\mu_{\max,a}\, S}{K_{S,a}+S} \qquad (12a)$$

$$\frac{1}{N_b}\frac{\mathrm{d}N_b}{\mathrm{d}t} = \mu_b = \frac{\mu_{\max,b}\, S}{K_{S,b}+S} \qquad (12b)$$

$$\frac{\mathrm{d}S}{\mathrm{d}t} = -\sum_{i=1}^{2} \frac{\alpha_i\, \mu_{\max,i}\, S}{K_{S,i}+S} N_i. \qquad (12c)$$

To calculate the competitive advantage during colonization we can assume that because the two populations are rare, cells initially grow exponentially with a constant growth rate and a negligible change in nutrient concentration over time:

$$\frac{1}{N_a}\frac{\mathrm{d}N_a}{\mathrm{d}t} = \mu_a = \frac{\mu_{\max,a}\, S}{K_{S,a}+S} \qquad (13a)$$

$$\frac{1}{N_b}\frac{\mathrm{d}N_b}{\mathrm{d}t} = \mu_b = \frac{\mu_{\max,b}\, S}{K_{S,b}+S} \qquad (13b)$$

$$\frac{\mathrm{d}S}{\mathrm{d}t} \approx 0. \qquad (13c)$$

Then, to calculate competitive advantage when rare, we can solve eqns. 13:

$$N_a(t) = N_a(0)e^{\mu_a t} \qquad (14a)$$

$$N_b(t) = N_b(0)e^{\mu_b t}, \qquad (14b)$$

where t is time (in days). Assuming $N_a(0) = N_b(0)$ (starting densities are equal, as in the experiments), we can define the competitive advantage (R) of species a relative to species b by taking the log of the ratio of their abundances at time t:

$$R = \ln\frac{N_a(t)}{N_b(t)} = \mu_a - \mu_b = S\left(\frac{\mu_{\max,a}(T)}{K_{S,a}(T)+S} - \frac{\mu_{\max,b}(T)}{K_{S,b}(T)+S}\right)t. \quad (15)$$

We now incorporate the effects of temperature change on the parameters μ_{max} and K_s of eqn. 15 to predict the effects of warming on competitive advantage.

Incorporating metabolic traits

Maximum growth rate μ_{max} is tightly coupled to the rate of net photosynthesis (Geider *et al.* 1998) and consequently, its temperature dependence is expected to be exponential up to a peak value (the optimum temperature), followed by a steeper exponential decline (Angilletta 2009; Padfield *et al.* 2016; Schaum *et al.* 2017). The temperature range of the initial exponential increase up to the optimum is the 'operational temperature range' (OTR) — the range most likely to be encountered by the population (Martin & Huey 2008; Pawar *et al.* 2016), and it can be described by

$$\mu_{\max,i} = B'_{0,i}\, m_i^{\beta} \exp\left(-\frac{E_{\mu,i}}{k}\left(\frac{1}{T} - \frac{1}{T_{\text{ref}}}\right)\right), \quad (16)$$

where $B'_{0,i}$ is a mass- and temperature-independent normalization constant, i.e., the value of $\mu_{\max,i}$ at a reference temperature T_{ref} (in K), $E_{\mu,i}$ is the activation energy (eV) that sets the relative rate of increase in $\mu_{\max,i}$ with temperature, k is the Boltzmann constant (eV·K^{-1}), T is temperature (K), m is cell mass (size), and β is the exponent of the scaling of growth rate with cell size (Eppley 1972; Kagami & Urabe 2001; Brown *et al.* 2004; DeLong *et al.* 2010). We define

$$B_{0,i} \equiv B'_{0,i} m_i^{\beta} \quad (17)$$

and therefore eqn. 16 becomes

$$\mu_{\max,i} = B_{0,i} \exp\left(-\frac{E_{\mu,i}}{k}\left(\frac{1}{T} - \frac{1}{T_{\text{ref}}}\right)\right). \quad (18)$$

Thus, interspecific differences in cell size m as well as the size scaling exponent β could contribute to differences in the species-specific normalization constants $B_{0,i}$, although the species used in the experiments were specifically chosen to have approximately similar cell sizes (Table S2A).

The shape of the relationship between K_S and temperature is less well known, with no comprehensive review on the subject. Several experimental studies found positive links between K_S and temperature in algae, plants and bacteria (Carter & Lathwell 1967; Shelef *et al.* 1970; Topiwala & Sinclair 1971; Thomas & Dodson 1974; Sawada *et al.* 1978; Mechling & Kilham 1982; Aksnes & Egge 1991; Sterner & Grover 1998), others found a hump-shaped relationship (Senft *et al.* 1981) or a negative relationship (Reay *et al.* 1999) while others found no evidence of temperature-dependence (Tilman *et al.* 1981; Ahlgren 1987). According to several theoretical studies, K_S is expected to increase with temperature (Goldman & Carpenter 1974; Aksnes & Egge 1991; Reuman *et al.* 2014). We assumed K_S to have a similar thermal response to $\mu_{\max}$, with the temperature dependence within the OTR of both $\mu_{\max}$ and K_S following the Boltzmann-Arrhenius equation,

$$K_{S,i} = K_{0,i} \exp\left(-\frac{E_{K,i}}{k}\left(\frac{1}{T} - \frac{1}{T_{\text{ref}}}\right)\right), \qquad (19)$$

where all parameters have the same meaning as in eqn. 16, and $K_{0,i}$ has been redefined to be a mass-scaling dependent normalization constant ($K_{0,i} \equiv K'_{0,i}\, m_i^{\beta}$). Our empirical results (see Figure 1 in the main text) support the use of the Boltzmann-Arrhenius function within the OTR. Comparing our empirical results to data on the same genus when available showed that our relationship was in accordance with previous experiments, with a positive temperature dependence in *Chlorella*, as found by Shelef *et al.* (1970), and no relationship with temperature in *Scenedesmus*, as found by Ahglren (1987). However, more empirical and theoretical work is needed to better understand the temperature-dependence of K_S.

Effects of metabolic traits on the competitive advantage

We can now substitute eqns. 18 and 19 into eqn. 15 to obtain the (relative) competitive advantage, R, of species a relative to species b in terms of differences in metabolic traits between the two species:

$$R = S\left(\frac{B_{0,a}\exp\left(-\frac{E_{\mu,a}}{k}\left(\frac{1}{T}-\frac{1}{T_{\text{ref}}}\right)\right)}{K_{0,a}\exp\left(-\frac{E_{K,a}}{k}\left(\frac{1}{T}-\frac{1}{T_{\text{ref}}}\right)\right)+S} - \frac{B_{0,b}\exp\left(-\frac{E_{\mu,b}}{k}\left(\frac{1}{T}-\frac{1}{T_{\text{ref}}}\right)\right)}{K_{0,b}\exp\left(-\frac{E_{K,b}}{k}\left(\frac{1}{T}-\frac{1}{T_{\text{ref}}}\right)\right)+S}\right)t. \qquad (20)$$

Thus the value of R depends on the differences in the competing species' metabolism, that is, on the differences in the respective parameters that define the temperature dependence of μ_{max} and K_S (B_0, E_μ, K_0, and E_K). When the parameters are equivalent in both species, $R = 0$, and both species are expected to be equally abundant at any time point t. When there are mismatches, $R \neq 0$, and the sign of R indicates which species has a competitive advantage: for $R > 0$, species a is expected to outnumber species b at time t, while the opposite is true for $R < 0$.

We can assess the relative importance of the metabolic traits characterising nutrient limited and resource saturated growth for predicting competitive advantage by comparing the full model for R (eq. 20) to a simplified version that assumes nutrient saturation (as $S \to \infty$):

$$R_\infty = \lim_{S\to\infty} R(S) = \left(\mu_{\text{max},a}(T) - \mu_{\text{max},b}(T)\right)t$$
$$= \left(B_{0,a}\exp\left(-\frac{E_{\mu,a}}{k}\left(\frac{1}{T}-\frac{1}{T_{\text{ref}}}\right)\right) - B_{0,b}\exp\left(-\frac{E_{\mu,b}}{k}\left(\frac{1}{T}-\frac{1}{T_{\text{ref}}}\right)\right)\right)t. \qquad (21)$$

In this case, species a will grow faster than species b if $R_\infty > 0$, and therefore if

$$\ln\frac{B_{0,a}}{B_{0,b}} > \frac{E_{\mu,a}-E_{\mu,b}}{k}\left(\frac{1}{T}-\frac{1}{T_{\text{ref}}}\right). \qquad (22)$$

Here, note that because the constants $B_{0,i}$ include the effects of size (eqn. 17), part of the mismatch in normalization constants is expected to come from differences in cell size. The trade-off between normalisation constants and activation energies here is explicit. At $T = T_{\text{ref}}$, the winner is entirely determined by the ratio in the normalisation constants (the right hand side of the inequality becomes zero). However, as T increases or decreases from T_{ref}, the relative importance of the activation energies increases, to the point that at a sufficiently large $|T - T_{\text{ref}}|$, the winner of the competition is entirely determined by the activation energy (see

Figure S1A below for an example). For narrower temperature ranges, such as those discussed in this study, the winner is determined by differences in both normalisation constants and activation energies.

A reversal in the competitive advantage R (a change in its sign) with temperature change is also possible, and can be determined numerically. For the nutrient saturated case, the temperature at which $R_\infty = 0$ is given by

$$T_{\text{rev}} = \frac{E_{\mu,a} - E_{\mu,b}}{k\left[\ln\frac{B_{0,a}}{B_{0,b}} + \frac{E_{\mu,a}-E_{\mu,b}}{kT_{\text{ref}}}\right]}. \quad (23)$$

Here, if there is a reversal, the species that wins at the higher temperature depends only on the difference in activation energies; for example, assuming a reversal takes place, if $E_{\mu,a} > E_{\mu,b}$, species a is expected to outcompete species b for $T > T_{\text{rev}}$.

Competitive advantage vs. competitive outcome

In line with the empirical scenario of co-invasion and our experimental setup, the above theory investigates how the exponential growth phase during colonisation determines competitive advantage between species competing for a single limiting resource. However, in the long run, and once populations reach high enough population densities, density dependence and intraspecific competition might be expected to play an increasingly important role. In the experiments, we inoculated the same (small) number of cells for both species at the start of the colonisation experiment, and then use the density of each species after 14 days to test the theory. The relative abundances of each species after 14 days indicates which had a competitive advantage after colonizing an empty environment. Because the initial competitive advantage is expected to result in an exponentially higher abundance of the competitively superior species (SI eq. 15 and 20), we expect that the advantage at 14 days will persist at the end of the experiment even if the species are no longer growing exponentially. This positive association between population growth rate and the long-term competitive outcome is consistent with theory and data, which suggest that equilibrium densities reflect the balance between density independent growth and density dependent regulation and thus higher intrinsic rates of increase tend to lead to higher equilibrium densities and competitive advantage (Mallet 2012). For reference, the median times to equilibrium density in the growth rate experiments were 11 and 9 days for very low (0.1 $\mu\text{mol}\cdot\text{L}^{-1}$ of phosphate) nutrient concentrations respectively for 15 and 25°C, 10.5 and 7 at low (1 $\mu\text{mol}\cdot\text{L}^{-1}$ of phosphate) nutrient concentrations, and 14.5 and 9 days at high (30 $\mu\text{mol}\cdot\text{L}^{-1}$ of phosphate) nutrient concentrations. Furthermore, we were also able to compare our theoretical predictions to results after 5 and 23 days of experiment, allowing us to check whether the assumption of the "carry-over signature" of competitive advantage beyond the exponential phase held true (Section S9).

Extensions to adaptive dynamics, and Tilman's* R* *theory

The full model (eqns. (11)) can be used to study more scenarios, including invasion while rare (where one species is introduced while the other is at its equilibrium density), and to explore longer-term adaptive (competitive) dynamics. In the invasion-while-rare scenario, Tilman *et al.* (1981) show that the species with the lowest equilibrium requirements of nutrients (S^*) will win, independent of the starting densities. In the model of eqn. (11), for instance,

$$S_i^* = \frac{D\,K_{S,i}}{\mu_{\max,i} - D}, \qquad (24)$$

so if a (resident) species is assumed to be at its equilibrium density, N^*, and a new (invading) species is introduced while rare, as long as $S_{\text{inv}}^* < S_{\text{res}}^*$, the invasion will be successful. The same argument is made within the adaptive dynamics framework, where S^* effectively represents the invasion fitness. If a mutant with a lower S^* is introduced to a population, it will successfully invade. With the temperature dependence of $\mu_{\max}$ and K_S made explicit using metabolic theory as we have done above, the conditions for invasion can be made explicitly temperature dependent and expressed in terms of mismatches between the resident and the invader population.

References

Ahlgren, G. (1987). Temperature Functions in Biology and Their Application to Algal Growth Constants. *Oikos*, 49, 177–190.

Aksnes, D.L. & Egge, J.K. (1991). A theoretical model for nutrient uptake in phytoplankton. *Marine Ecology Progress Series*, 70, 65–72.

Angilletta, M.J. (2009). *Thermal Adaptation: A Theoretical and Empirical Synthesis*. Oxford University Press.

Brown, J.H., Gillooly, J.F., Allen, A.P., Savage, V.M. & West, G.B. (2004). Toward a metabolic theory of ecology. *Ecology*, 85, 1771–1789.

Bulgakov, N.G. & Levich, A.P. (1999). The nitrogen : Phosphorus ratio as a factor regulating phytoplankton community structure : Nutrient ratios. *Archiv für Hydrobiologie*, 146, 3–22.

Carter, O.G. & Lathwell, D.J. (1967). Effects of Temperature on Orthophosphate Absorption by Excised Corn Roots. *Plant Physiol.*, 42, 1407–1412.

DeLong, J.P., Okie, J.G., Moses, M.E., Sibly, R.M. & Brown, J.H. (2010). Shifts in metabolic scaling, production, and efficiency across major evolutionary transitions of life. *PNAS*, 107, 12941–12945.

Eppley, R.W. (1972). Temperature and phytoplankton growth in the sea. *Fishery Bulletin*, 70, 1063–1085.

Geider, R.J., MacIntyre, H.L. & Kana, T.M. (1998). A dynamic regulatory model of phytoplanktonic acclimation to light, nutrients, and temperature. *Limnology and Oceanography*, 43, 679–694.

Goldman, J.C. & Carpenter, E.J. (1974). A kinetic approach to the effect of temperature on algal growth. *Limnology and Oceanography*, 19, 756–766.

Kagami, M. & Urabe, J. (2001). Phytoplankton growth rate as a function of cell size: an experimental test in Lake Biwa. *Limnology*, 2, 111–117.

Mallet, J. (2012). The struggle for existence: how the notion of carrying capacity, K, obscures the links between demography, Darwinian evolution, and speciation. *Evol Ecol Res*, 14, 627–665.

Martin, T.L. & Huey, R.B. (2008). Why "Suboptimal" Is Optimal: Jensen's Inequality and Ectotherm Thermal Preferences. *The American Naturalist*, 171, E102–E118.

Mechling, J.A. & Kilham, S.S. (1982). Temperature Effects on Silicon Limited Growth of the Lake Michigan Diatom Stephanodiscus Minutus (bacillariophyceae)1. *Journal of Phycology*, 18, 199–205.

Padfield, D., Yvon-Durocher, G., Buckling, A., Jennings, S. & Yvon-Durocher, G. (2016). Rapid evolution of metabolic traits explains thermal adaptation in phytoplankton. *Ecol Lett*, 19, 133–142.

Pawar, S., Dell, A.I., Savage, V.M. & Knies, J.L. (2016). Real versus Artificial Variation in the Thermal Sensitivity of Biological Traits. *The American Naturalist*, 187, E41–E52.

Reay, D.S., Nedwell, D.B., Priddle, J. & Ellis-Evans, J.C. (1999). Temperature Dependence of Inorganic Nitrogen Uptake: Reduced Affinity for Nitrate at Suboptimal Temperatures in Both Algae and Bacteria. *Appl. Environ. Microbiol.*, 65, 2577–2584.
Reuman, D.C., Holt, R.D. & Yvon-Durocher, G. (2014). A metabolic perspective on competition and body size reductions with warming. *J Anim Ecol*, 83, 59–69.
Sawada, T., Chohji, T. & Kuno, S. (1978). Kinetic Analysis of Unbalanced Bacterial Growth in Temperature Shift. In: *Chemical Reaction Engineering—Houston* (eds. Weekman, V.W. & Luss, D.). AMERICAN CHEMICAL SOCIETY, WASHINGTON, D. C., pp. 163–172.
Schaum, C.-E., Barton, S., Bestion, E., Buckling, A., Garcia-Carreras, B., Lopez, P., *et al.* (2017). Adaptation of phytoplankton to a decade of experimental warming linked to increased photosynthesis. *Nature Ecology & Evolution*, 1, 0094.
Senft, W.H., Hunchberger, R.A. & Roberts, K.E. (1981). Temperature Dependence of Growth and Phosphorus Uptake in Two Species of Volvox (volvocales, Chlorophyta). *Journal of Phycology*, 17, 323–329.
Shelef, G., Oswald, J. & Golueke, C. (1970). Assaying algal growth with respect to nitrate concentration by a continuous flow turbidostat. *Proc. Int. Conf. Water. Pollut. Rees.*, 3–25.
Sterner, R.W. & Grover, J.P. (1998). Algal growth in warm temperate reservoirs: kinetic examination of nitrogen, temperature, light, and other nutrients. *Water Research*, 32, 3539–3548.
Thomas, W.H. & Dodson, A.N. (1974). Effect of interactions between temperature and nitrate supply on the cell-division rates of two marine phytoflagellates. *Mar. Biol.*, 24, 213–217.
Tilman, D. (1977). Resource Competition between Plankton Algae: An Experimental and Theoretical Approach. *Ecology*, 58, 338–348.
Tilman, D. (1981). Tests of Resource Competition Theory Using Four Species of Lake Michigan Algae. *Ecology*, 62, 802–815.
Tilman, D., Mattson, M. & Langer, S. (1981). Competition and nutrient kinetics along a temperature gradient: An experimental test of a mechanistic approach to niche theory. *Limnol. Oceanogr.*, 26, 1020–1033.
Topiwala, H. & Sinclair, C.G. (1971). Temperature relationship in continuous culture. *Biotechnol. Bioeng.*, 13, 795–813.

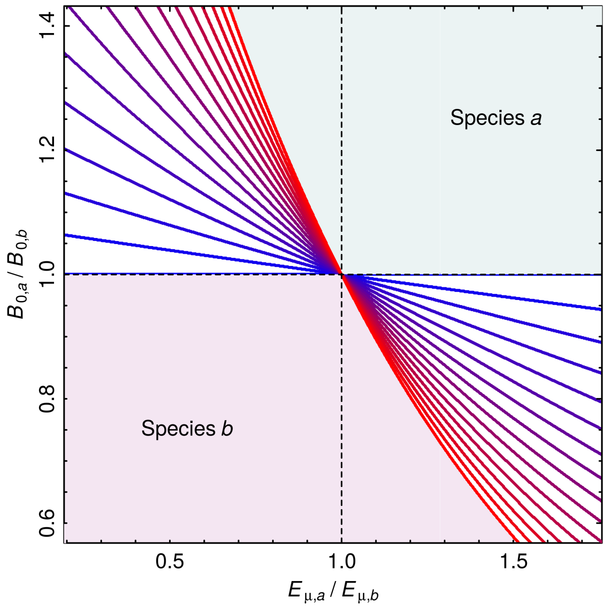

Figure S1A. Contour lines illustrating the competitive advantage for a range of parameter combinations, assuming nutrient saturation ($\boldsymbol{R_\infty}$). The colour of the lines correspond to different temperatures, ranging from 15°C for the blue line, to 30°C for the red line. For example, for $E_{\mu,a}/E_{\mu,b}$= 1 and $B_{0,a}/B_{0,b}$ = 0.8, species b grows faster than species a, but for $E_{\mu,a}/E_{\mu,b}$ = 0.5 and $B_{0,a}/B_{0,b}$ = 1.2, which species grows faster depends on the temperature. Here, $B_{0,b} = 1$, $E_{\mu,b} = 0.55$, and $T_{\text{ref}} = 15$°C. Therefore, at $T = 15$°C, which species wins is determined by $B_{0,a}/B_{0,b}$ (the blue line is horizontal and insensitive to the ratio in activation energies), while as temperatures move further away from T_{ref}, the ratio of activation energies becomes increasingly important in determining the competitive advantage. As temperature increases beyond the range shown here, the lines become increasingly vertical, and as a result, insensitive to the ratio of normalization constants.

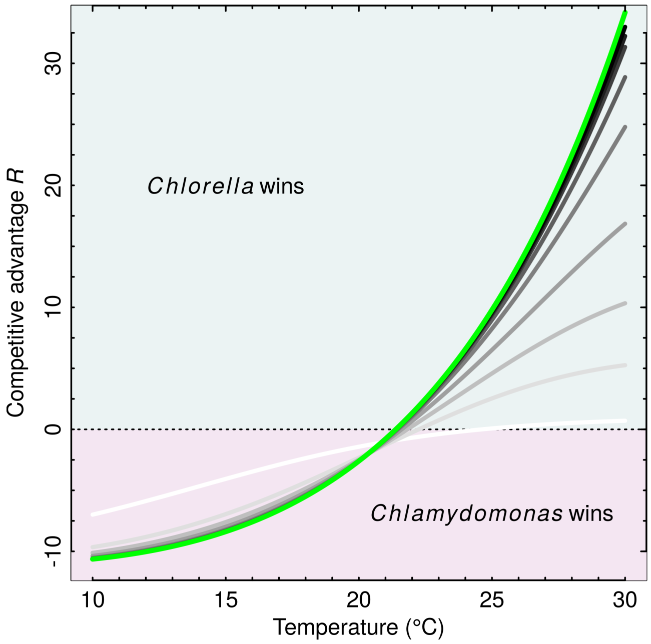

Figure S1B. Example of a reversal in the competitive advantage, R, across a temperature range. The green line is for nutrient saturated conditions (R_{∞}), and the grayscale lines are for different nutrient concentrations, ranging from $S = 0.1$ μmol·L^{-1} for the light gray line, to 50 μmol·L^{-1} for the black line. The example uses parameters for *Chlorella* and *Chlamydomonas*, where $R > 0$ means *Chlorella* has a competitive advantage over *Chlamydomonas*.

S2. Experimental design

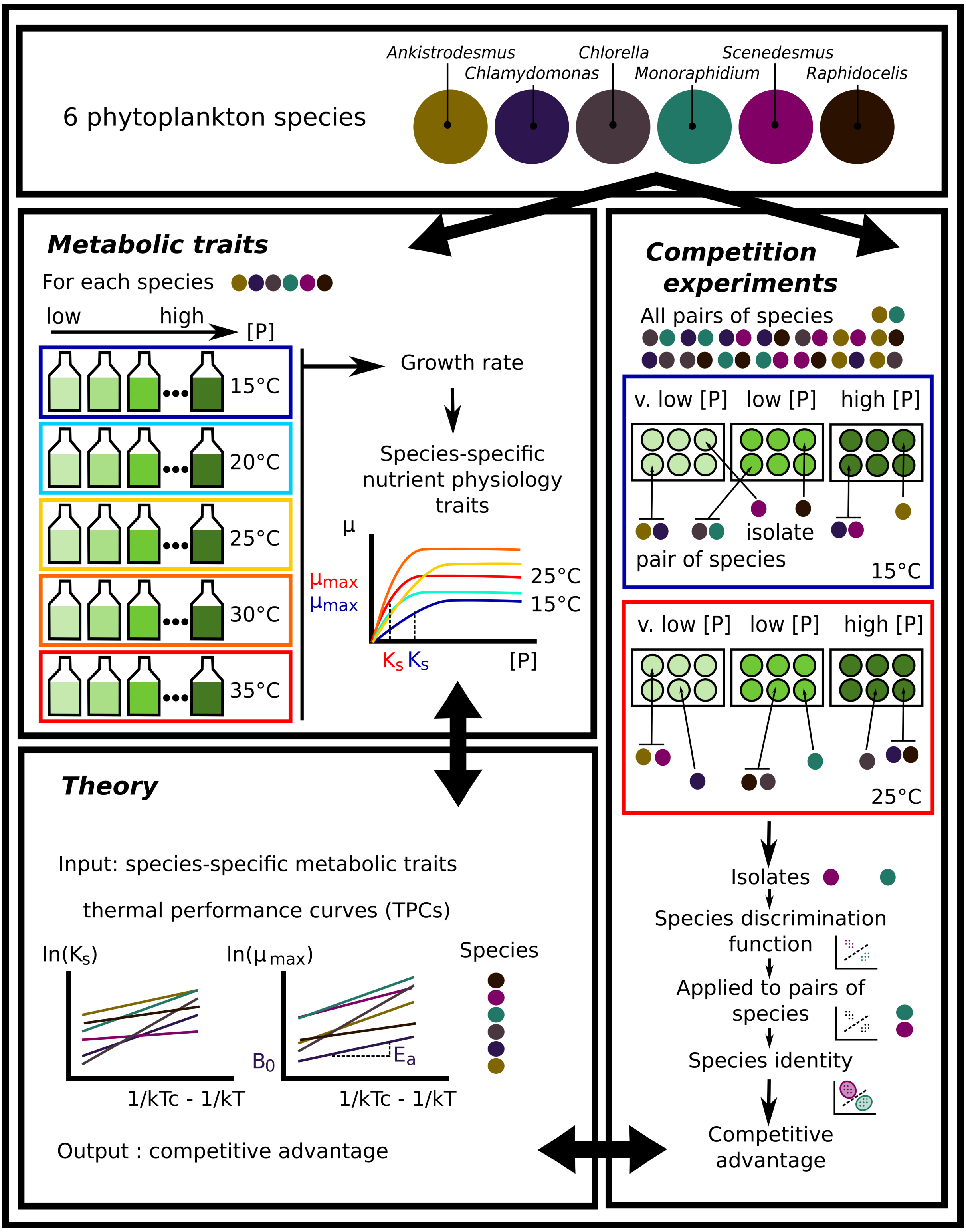

Figure S2A. Flow chart of the experimental design

Table S2A. Detailed information about the six species.
The species were ordered from the Culture Collection of Algae and Protozoa (www.ccap.ac.uk). Cell diameters are calculated from microscopy pictures as the average of the longest and shortest diameter of the cell over 30 cells.

**Species name**	**Class**	**Order**	**Strain**	**Origin**	**Mean cell diameter (µm)**
Ankistrodesmus nannoselene Skuja (1948)	Chlorophyceae	Sphaeropleales	CCAP 202/6A	Siggeforsajon, Sweden (1948)	2.8
Chlamydomonas moewusii Gerlof (1940)	Chlorophyceae	Chlamydomonadales	CCAP 11/5A	Freshwater	8.1
Chlorella sorokiniana Shihira& Krauss (1965)	Trebouxiophyceae	Chlorellales	CCAP 211/8K	Austin, Texas, USA (1953)	4.2
Monoraphidium minutum(Nägeli) Komarkova-Legnerova (1969)	Chlorophyceae	Sphaeropleales	CCAP 278/3	Texas, USA	4.7
Scenedesmus obliquus (Turpin) Kützing (1833)	Chlorophyceae	Sphaeropleales	CCAP 276/3B	Lund, Sweden (1939)	7.1
Raphidocelis subcapitata (formerly *Selenastrum capricornutum)* Printz (1913)	Chlorophyceae	Sphaeropleales	CCAP 278/4	Akershus, Norway (1959)	5.8

Table S2B. Phosphate concentration levels for each solution, in µmol·L^{-1} and µg·L^{-1}.
We created 13 solutions of different phosphate concentrations ranging from 0.01 µmol·L^{-1} of phosphate to 50 µmol·L^{-1} of phosphate by mixing different amounts of COMBO medium without potassium phosphate dibasic (P- COMBO) and normal COMBO medium (P+ COMBO) in 40 mL tissue culture flasks. We used a modified version of the standard COMBO medium without animal trace solution in which we increased the fraction of carbonate by adding 10 mL of a stock solution of 55.8 g·L^{-1} of sodium bicarbonate to maintain a DIC of more than 6.6 mmol·L^{-1} in order to prevent carbon limitation, which maintained a C:N:P ratio of 132:20:1 in the P+ COMBO solution, above the Redfield ratio of 106:16:1.

Phosphate concentration (µmol·L^{-1})	50	40	30	20	10	8	6	4	2	1	0.5	0.1	0.01
Phosphate concentration (µg·L^{-1})	4750	3800	2850	1900	950	760	570	380	190	95	47.5	9.5	0.95
Amount of P+ COMBO (mL)	40	32	24	16	8	6.4	4.8	3.2	1.6	0.8	0.4	0.08	0.008
Amount of P- COMBO (mL)	0	8	16	24	32	33.6	35.2	36.8	38.4	39.2	39.6	40	40

S3. Discrimination between species in the competition experiment

To investigate the joint effects of temperature and phosphate availability on competition, we competed all species in all pairwise combinations (15 pairs) at two temperatures (15 and 25°C; low temperature and a temperature close to the optimum for most species, Fig. 1) and three phosphate concentrations: one saturating [30 $\mu mol \cdot L^{-1}$] and two limiting [1 $\mu mol \cdot L^{-1}$ and 0.1 $\mu mol \cdot L^{-1}$] concentrations, chosen from the Monod curves, Fig. 1), with each replicated 6 times (Fig. S2A), amounting to 540 samples. Along with the pairwise competition trials, we grew all 6 species in monoculture at the two temperatures and three nutrient levels. This was to train the discrimination algorithm used to separate cells from different species in the competition trial. The monoculture trials were divided into two subsets, one to train the cell discrimination algorithm, which was replicated 3 times per temperature and nutrient levels, and a testing subset used to test the accuracy of the cell discrimination algorithm, which was replicated 6 times. This testing subset was also used to calculate total yield in monoculture to compare it to yield in biculture (see Section S8). The competition experiments were carried out in two batches, a first batch in June 2016 for the 30 $\mu mol \cdot L^{-1}$ and 1 $\mu mol \cdot L^{-1}$ P and a second batch in October 2017 for the very low nutrient concentration (0.1 $\mu mol \cdot L^{-1}$ P). This second batch was added to further explore nutrient limited competition as the Monod curves indicated that 1 $\mu mol \cdot L^{-1}$ P was above the half-saturating constant for some species, particularly at low temperatures (see Table S4B). The competition experiments were carried out in 24 well plates filled with 2 mL of media and inoculated with 100 $cells \cdot mL^{-1}$ of each species, ensuring that the increase in phosphate concentration due to the inoculum volume (1 µL of sample at $2x10^5$ $cells.mL^{-1}$) or due to potential storages of phosphate in the cells was minimal (0.025 $\mu mol \cdot L^{-1}$ P). Plates were covered with AeraSeal™ breathable membrane, minimising evaporation and contamination but allowing gas exchange. The competition plates were incubated in the same way as described for the monoculture growth curves. At day 5, 14 and 23, a 200 µL sample was taken and preserved as described in the metabolic traits section. Cell density was determined by flow cytometry on the slow flux setting (14 µL·min), counting 20 µL per sample. A preliminary test measuring twice the same sample on 54 samples (6 species x 9 replicates) gave a mean variation between cell counts of 9%. We focus on the results from day 14 in the main results and for the description of the discrimination algorithm method; however, rerunning the analyses using day 5 or day 23 gave qualitatively similar results (see Supplementary Section S9 for results on these two other days).

FSC files returned by the flow cytometer were read with the Bioconductor 'FlowCore' package in R, returning side scatter (SSC), forward scatter (FSC), green fluorescence (FL1), orange fluorescence (FL2), red fluorescence (FL3), and blue fluorescence (FL4) values that could be used to define species morphology and pigment composition and thus discriminate between species in the pairwise competition assays. We first filtered the data to remove noise by removing every data point where either ln(FSC.H)<10.3, ln(SSC.H)<3 or ln(FL3.H)<1.5, which are below minimum values observed for life cells of all 6 species. The training dataset was used to determine discrimination functions between pairs of species. We used the data collected at day 14 to train the discrimination algorithm, except for the P = 0.1 $\mu mol \cdot L^{-1}$ dataset where we pooled all of the data together to get a greater discrimination power as cell densities were very low under these conditions. We first removed outliers from this dataset by manually inspecting FSC.H by FL3.H clustering plots and choosing visual thresholds for these two values for each species. We then applied 3 different procedures to discriminate between pairs of species for each temperature and phosphate level: a linear discriminant analysis with the 'lda' function from the 'MASS' package, a random forest analysis with the 'randomForest' function from the 'randomForest' package, and a recursive partitioning and regression tree analysis with the

‘rpart’ function from the ‘rpart’ package. These analyses were performed using the natural logarithm of the 10 variables returned by the flow cytometer (that is FSC.H, FSC.A, SSC.H, SSC.A, FL1.H, FL1.A, FL2.H, FL2.A, FL3.H, FL3.A, FL4.H and FL4.A, .H standing for height and .A for area), on each of the 15 pairs of species for each combination of temperature and phosphate level, except for the P = 0.1 $\mu mol \cdot L^{-1}$ dataset, where we pooled all temperatures together to get a greater discrimination power. These different discriminant functions were then applied to the testing dataset to test the accuracy of the predictions for the different discriminant methods. For each pair of species, we used the training set to create *in silico* competition experiments where 100% of the cells would pertain to one of the species. We applied the discrimination algorithm and calculated the percentage of times where a cell was wrongly attributed to the other species. We then chose the method that gave the maximum level of accuracy to apply to the competition dataset (Fig. S3A). The best method was the linear discriminant analysis, which gave 78% accuracy (Table S3A). However, we checked that the results were robust to the statistical method used to discriminate between species (Section S6 in SI).

After determining species identity for each sample, we computed cell density and calculated the competitive advantage R of species a relative to species b by taking the ln ratio of their densities ($cells \cdot mL^{-1}$) at time t, adding 1 to each species density for instances when one species became locally extinct (i.e., when density = 0). We also computed a binary competitive advantage where species a was competitively dominant when $R > 0$ and vice versa. Because the efficacy of the discrimination algorithm depends on having a sufficient quantity of data with which to assign identities, we set a minimum threshold of N_{tot} = 500 $cells \cdot mL^{-1}$. This led us to discard 171 replicates out of 540 for day 14. Furthermore, in comparisons with the model, we removed 9 replicates for which the observed $R = 0$, because the model necessarily predicts a non-zero R (traits characterising the TPCs for μ_{max} and K_S were never identical for any species pair).

Table S3A. Performance of the discrimination algorithms at day 14.
LDA: linear discriminant analysis, Random Forest analysis, RPART: recursive partitioning and regression tree. Summaries by (a) species for all nutrient and thermal conditions, (b) pairs of species for all nutrient and thermal conditions, (c) phosphate and nutrient conditions for all pairs of species.

a

**Species**	**LDA**	**Randomforest**	**RPART**
Ankistrodesmus	0.84	0.78	0.67
Chlamydomonas	0.89	0.90	0.81
Chlorella	0.74	0.77	0.62
Monoraphidium	0.76	0.74	0.64
Scenedesmus	0.79	0.76	0.63
Raphidocelis	0.65	0.66	0.50
**Mean**	**0.78**	**0.77**	**0.65**

b

**Pair of species**	**LDA**	**Randomforest**	**RPART**
Ankistrodesmus-Chlamydomonas	0.96	0.98	0.93
Ankistrodesmus-Chlorella	0.91	0.65	0.52
Ankistrodesmus-Monoraphidium	0.84	0.76	0.72
Ankistrodesmus-Scenedesmus	0.90	0.89	0.73
Ankistrodesmus-Raphidocelis	0.67	0.61	0.46
Chlamydomonas-Chlorella	0.91	0.94	0.83
Chlamydomonas-Monoraphidium	0.93	0.94	0.86
Chlamydomonas-Scenedesmus	0.82	0.85	0.71
Chlamydomonas-Raphidocelis	0.80	0.81	0.76
Chlorella-Monoraphidium	0.62	0.72	0.59
Chlorella-Scenedesmus	0.80	0.81	0.66
Chlorella-Raphidocelis	0.57	0.72	0.49
Monoraphidium-Scenedesmus	0.82	0.69	0.63
Monoraphidium-Raphidocelis	0.57	0.62	0.37
Scenedesmus-Raphidocelis	0.60	0.54	0.44
**Mean**	**0.78**	**0.77**	**0.65**

c

Temperature	Nutrient	LDA	Random forest	RPART
15	0.1	0.62	0.62	0.58
15	1	0.79	0.68	0.64
15	30	0.85	0.8	0.76
25	0.1	0.63	0.71	0.62
25	1	0.7	0.69	0.68
25	30	0.64	0.66	0.62
**Mean**		**0.71**	**0.69**	**0.65**

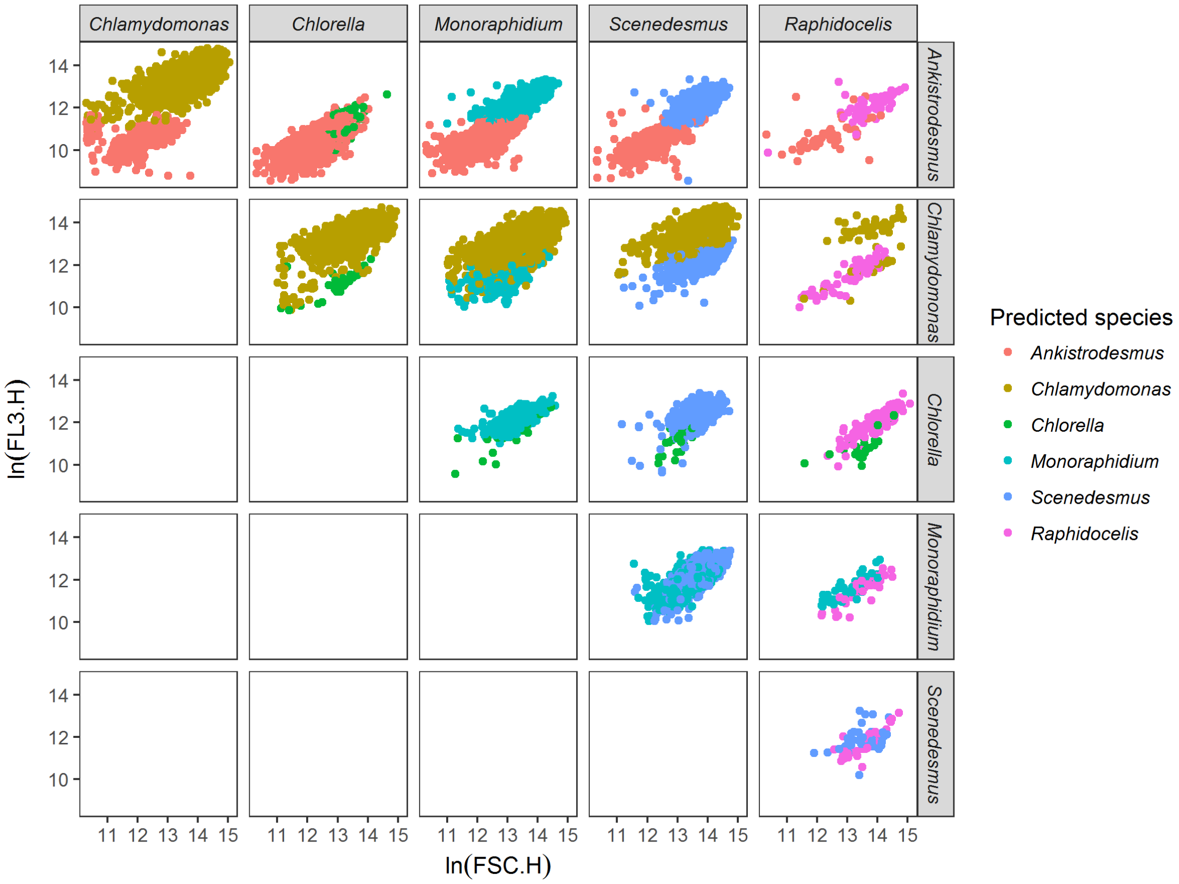

Figure S3A. Example of discrimination between species among pairs of species. We here show species grown at 15°C in saturating nutrient conditions (P = 30 µmol·L^{-1}) after 14 days of experiment. Each dot represents a cell, here mapped on FSC.H (size proxy) and FL3.H (chlorophyll a proxy) characteristics from the flow cytometer. Colours represent the species predicted by the discrimination algorithm. The discrimination algorithm is a linear discriminant analysis trained with flow cytometer data (FSC.H, FSC.A, SSC.H, SSC.A, FL1.H, FL1.A, FL2.H, FL2.A, FL3.H, FL3.A, FL4.H, and FL4.A) from the species grown in isolates at the same temperature and nutrient conditions. For example, *Chlamydomonas* has a competitive advantage over *Chlorella* in these nutrient and temperature conditions (there are more *Chlamydomonas* cells).

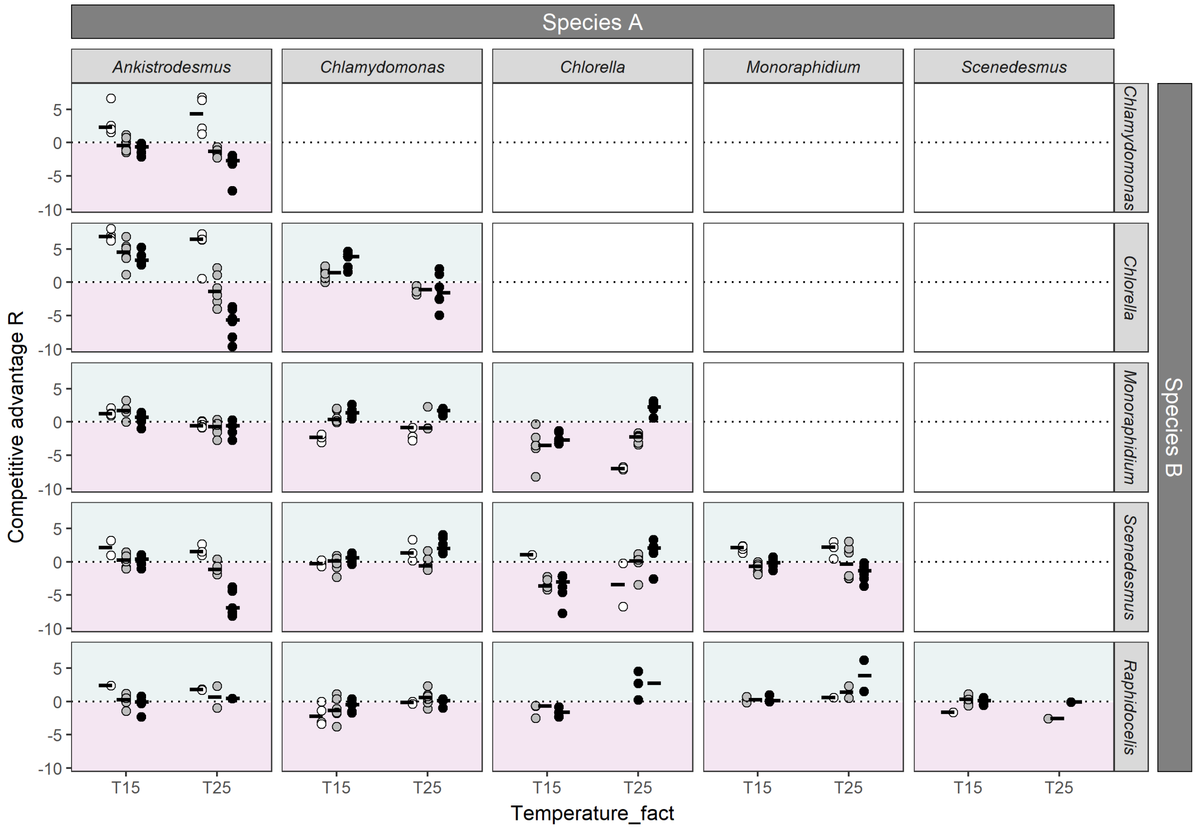

Figure S3B. Competition outcomes at day 14.

For each pair of species, the competitive advantage *R*. Circle colour represents the nutrient conditions of the trial; white circles: very low nutrient concentration (0.1 µmol·L^{-1} of phosphate); grey circles: low nutrient concentration (1 µmol·L^{-1} of phosphate); black circles: saturated nutrient solution (30 µmol·L^{-1} of phosphate). Points represent the values of each of the 6 replicates per condition. Note that when the total cell density did not reach a threshold value of 500 cells·mL^{-1}, the replicates were discarded (see Methods), thus for some of the very low nutrient concentration cases no replicates were kept for a given pair. The segment represents the median of the replicates. The dotted lines represent the situation where there is no competitive advantage between the species ($N_A = N_B$). The area above the line shows an advantage for species A (turquoise colour), while area below the line shows and advantage for species B (pink colour). We can see for instance that for the *Ankistrodesmus-Chlorella* pair of species, *Ankistrodesmus* dominates at low temperatures for all nutrient conditions while *Chlorella* dominates at high temperatures, particularly at high nutrient conditions.

S4. Temperature dependence of the Monod model parameters

Table S4A. Metabolic traits for each alga.

Normalization constants (B_0 and K_0 resp. for $\mu_{\max}$ and K_S) and activation energies (E_μ and E_K resp. for $\mu_{\max}$ and K_S) derived from a Boltzmann-Arrhenius model fit on ln scales using nonlinear least squares to the values of $\mu_{\max}$ and K_S for all replicates, for temperatures between 15 and 25°C, and using a reference temperature $T_{\mathrm{ref}} = 15°\mathrm{C}$ (estimates ± SE). Note that for some replicates, the Monod model gave $K_S = 0$. Because the Boltzmann-Arrhenius model was fit on ln scales and to avoid infinite values when applying the logarithm to these values, these were set to the minimum quantity of nutrients in the experiment, that is $K_S = 0.001$.

**Species**	K_S		$\mu_{\max}$	
	$\ln B_0$	E_K	$\ln B_0$	E_μ
Ankistrodesmus	-6.49 ± 0.51	3.26 ± 0.59	-0.39 ± 0.04	0.27 ± 0.05
Chlamydomonas	-2.47 ± 0.63	0.96 ± 0.72	0.15 ± 0.07	0.16 ± 0.08
Chlorella	-2.71 ± 0.19	1.49 ± 0.22	-0.58 ± 0.07	0.99 ± 0.08
Monoraphidium	-3.44 ± 0.73	1.47 ± 0.83	-0.54 ± 0.09	0.59 ± 0.10
Scenedesmus	-1.30 ± 0.46	0.00 ± 0.52	0.22 ± 0.07	0.00 ± 0.08
Raphidocelis	-1.89 ± 0.52	2.30 ± 0.60	-0.50 ± 0.17	0.90 ± 0.19

Table S4B: Half-saturation constants (K_S) and degree of nutrient saturation.

Percentage of $\mu_{\max}$ at the low and very low experimental nutrient concentrations for the competition experiment calculated from values in Table S4A. For each species, this indicates whether species are close to nutrient saturation at the experimental temperature and phosphate concentration chosen for the competition experiment.

**Species**	K_S		**Growth at 1 µmol·L^{-1} as % of $\mu_{\max}$**		**Growth at 0.1 µmol·L^{-1} as % of $\mu_{\max}$**	
	**15°C**	**25°C**	**15°C**	**25°C**	**15°C**	**25°C**
Ankistrodesmus	0.002	0.124	100%	89%	99%	44%
Chlamydomonas	0.085	0.309	91%	76%	54%	24%
Chlorella	0.067	0.500	94%	67%	60%	16%
Monoraphidium	0.032	0.233	97%	81%	76%	30%
Scenedesmus	0.274	0.274	79%	79%	27%	27%
Raphidocelis	0.151	3.386	87%	23%	40%	3%

Table S4C. Results from the GAMs of $\ln(\mu_{max})$ **as a function of temperature**
For each species. See Fig. 1 for the representation of the GAMs.

Species	edf	F	p-value	R^2
Ankistrodesmus	2	8.33	0.005**	0.51
Chlamydomonas	2	3.96	0.048*	0.30
Chlorella	2	113.6	>0.001***	0.94
Monoraphidium	2	70.4	>0.001***	0.91
Scenedesmus	2	0.34	0.716	-0.10
Raphidocelis	2	9.60	0.003**	0.56

Table S4D. Results from the GAMs of $\ln(K_s)$ **as a function of temperature**
For each species. See Fig. 1 for the representation of the GAMs.

Species	edf	F	p-value	R^2
Ankistrodesmus	2	31.6	>0.001***	0.81
Chlamydomonas	2	4.39	0.037*	0.33
Chlorella	2	27.5	>0.001***	0.79
Monoraphidium	2	6.21	0.014*	0.43
Scenedesmus	2	1.49	0.265	0.06
Raphidocelis	2	12.28	0.001**	0.62

S5. Significance of competitive advantage predicted by the model.

To quantify the significance of the theory's ability to predict competitive advantage, we ran the analysis 10,000 times, sampling the values of B_0, E_μ, K_0, and E_K independently, with replacement, from the pool of available values. The analysis produced 10,000 sets of predictions, and therefore 10,000 proportions of competitive advantages correctly predicted (e.g., Fig. S5A). The proportion of runs that correctly predicted a greater number of competitive advantages than the real parameter values are then given as the *P* values in Table 1. Therefore, *P*=0.05 means that 500 out of 10,000 random parameter combinations correctly predicted a greater proportion of competitive advantages.

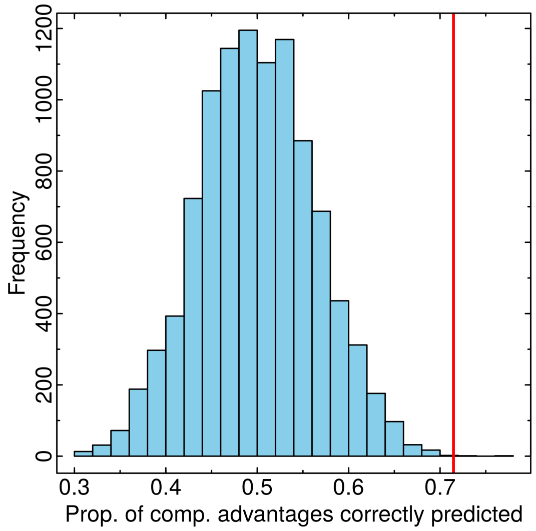

Figure S5A. Histogram of proportions of competitive advantages correctly predicted for 10,000 random parameter combinations.

The real parameters correctly predicted the competitive advantage in 72% of the competitions (red line), and 2 of the 10,000 random parameter combinations produced a greater predictive power (>72% of correct predictions; runs to the right of the red line).

S6. Robustness of the results to different statistical methods

We used three different methods of discrimination to determine the number of cells from each species, a linear discriminant analysis, a random forest analysis and a recursive partitioning and regression tree (rpart, see Section S3 in SI). Because the linear discriminant analysis was found to have the best predictive power overall (Table S3A), we used this method throughout the manuscript. However, we tested whether our results were robust to the method of species discrimination by comparing results from the competition model to predictions using the random forest analysis and the rpart discrimination method (Table S6A and S6B). The results were similar, with a lower predictive power of each variable and of the model due to the lower discrimination power of the two methods, but no significant discrepancies between species and temperature and nutrient conditions.

Table S6A. Proportion of competitive advantages correctly predicted by theory using the random forest discrimination method at day 14.

Analogous to Table 1 in the main text, but using the random forest discrimination method.

	R_∞		R		N
Full dataset					
	0.60	(0.014)	0.70	(0.000)	365
By temperature					
$T = 15°C$	0.66	(0.054)	0.72	(0.003)	192
$T = 25°C$	0.54	(0.138)	0.68	(0.005)	173
By nutrient					
[P] = 0.1 μmol·L^{-1}	0.33	(0.786)	0.78	(0.051)	69
[P] = 1 μmol·L^{-1}	0.59	(0.136)	0.62	(0.055)	151
[P] = 30 μmol·L^{-1}	0.74	(0.005)	0.74	(0.005)	145
By species					
Ankistrodesmus	0.64	(0.015)	0.80	(0.000)	137
Chlamydomonas	0.59	(0.012)	0.66	(0.014)	140
Chlorella	0.75	(0.026)	0.83	(0.003)	119
Monoraphidium	0.55	(0.151)	0.69	(0.010)	134
Scenedesmus	0.57	(0.079)	0.62	(0.031)	126
Raphidocelis	0.46	(0.752)	0.54	(0.239)	74

Table S6B. Proportion of competitive advantages correctly predicted by theory using the rpart discrimination method at day 14.
Analogous to Table 1 in the main text, but using the rpart discrimination method.

	R_{∞}		R		N
Full dataset					
	0.60	(0.026)	0.68	(0.001)	367
By temperature					
$T = 15°C$	0.64	(0.083)	0.71	(0.012)	193
$T = 25°C$	0.55	(0.188)	0.66	(0.017)	174
By nutrient					
[P] = 0.1 $\mu mol \cdot L^{-1}$	0.37	(0.741)	0.73	(0.092)	71
[P] = 1 $\mu mol \cdot L^{-1}$	0.58	(0.176)	0.62	(0.073)	150
[P] = 30 $\mu mol \cdot L^{-1}$	0.73	(0.014)	0.73	(0.014)	146
By species					
Ankistrodesmus	0.65	(0.022)	0.77	(0.000)	137
Chlamydomonas	0.56	(0.081)	0.63	(0.047)	140
Chlorella	0.76	(0.020)	0.84	(0.003)	119
Monoraphidium	0.56	(0.131)	0.67	(0.023)	135
Scenedesmus	0.56	(0.124)	0.60	(0.055)	126
Raphidocelis	0.45	(0.598)	0.55	(0.287)	77

S7. Quantitative relationship between theoretical and experimental outcomes

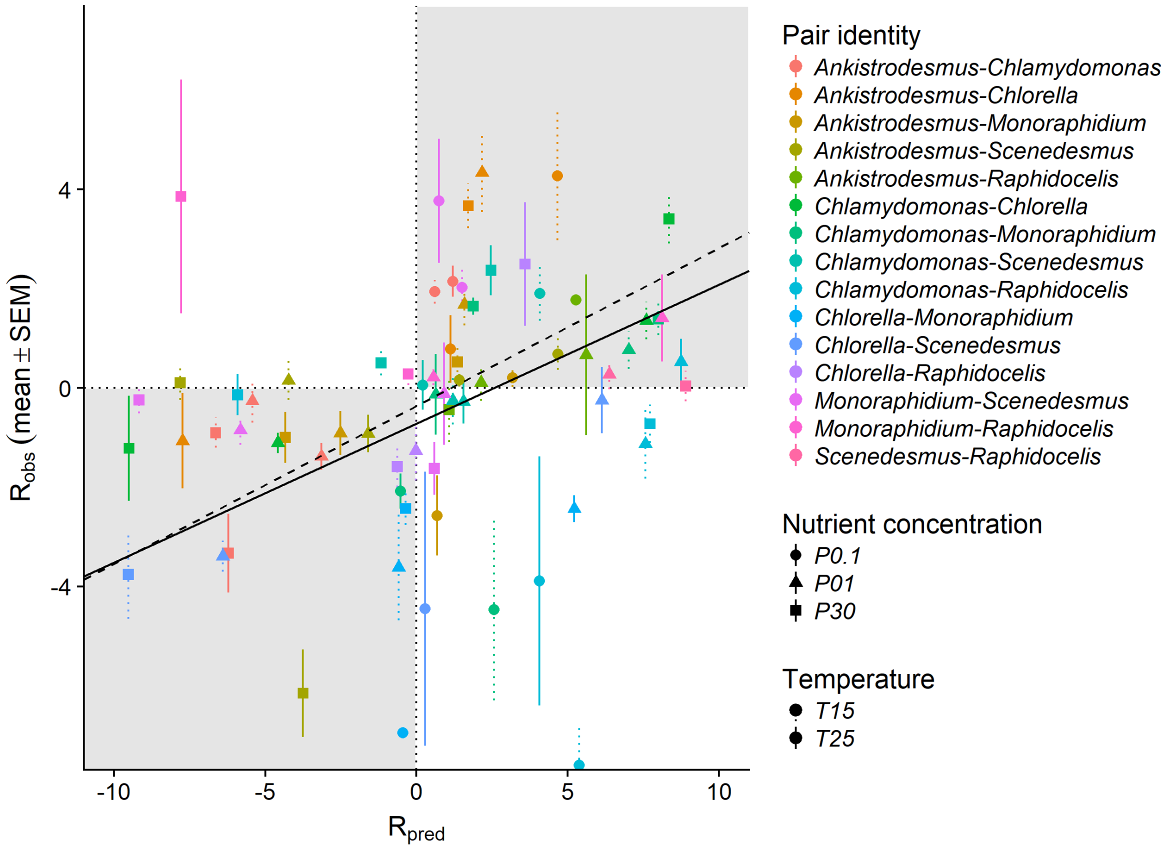

Figure S7A. Correlation between the observed and predicted competitive advantage at day 14. Different species pairs are in different colours, circles are for very low nutrient concentration, triangle for low nutrient concentration and squares for high nutrient concentrations, and the type of the standard error line stands for the temperature (dotted for low temperature, solid for high temperature). Most of the binary experimental outcomes (sign of observed *R*) fall in the same region (grey rectangles) as the binary theoretical outcomes (sign of predicted *R*). The full line represents the results of a linear mixed model of observed *R* as a function of predicted *R* as a fixed effect plus pair ID, temperature and nutrients as random intercepts on the whole dataset, while the dashed line represents the results from the same model but excluding pairs involving *Raphidocelis* (see Table S7A and Table S7B for details about the model).

Table S7A. Results from the linear mixed model investigating observed *R* as a function of predicted *R* at day 14.

Model includes predicted *R* as a fixed effect plus pair ID, temperature and nutrients as random intercepts with lmer function from lme4 package (Robs ~ Rpred + (1|temperature) + (1|nutrient) + (1|species pair)). N = 369.

Factor	Estimate	SE	t-value	χ^2 statistics	R^2
Fixed effect					***marginal R^2***
R_{pred}	0.29	0.02	13.04	$\chi^2 = 150$, $p < 2e^{-16}$	0.29
Random effect	**Variance**				***conditional R^2***
Temperature	0.08				0.54
Nutrient	0.10				
Pair identity	2.04				
Residual	4.21				

Table S7B. Results from the linear mixed model investigating observed *R* as a function of predicted *R* at day 14 excluding pairs involving *Raphidocelis.*

Model includes predicted *R* as a fixed effect plus pair ID, temperature and nutrients as random intercepts with lmer function from lme4 package (Robs ~ Rpred + (1|temperature) + (1|nutrient) + (1|species pair)). N = 292.

Factor	Estimate	SE	t-value	χ^2 statistics	R^2
Fixed effect					***marginal R^2***
R_{pred}	0.34	0.02	14.33	$\chi^2 = 205$, $p < 2e^{-16}$	0.34
Random effect	**Variance**				***conditional R^2***
Temperature	0.43				0.62
Nutrient	0.08				
Pair identity	2.65				
Residual	4.09				

Table S7C. Link between observed and predicted *R* at day 14 by species.

Results from a mixed effect model of Robs ~ Rpred + (1|temperature)+(1|nutrient)+(1|species pair) for each subset of competitions.

Species	Fixed R_{pred} effect estimate	SD	t-value	Marginal R^2	Conditional R^2	N
Ankistrodesmus	0.35	0.05	6.94	0.27	0.63	138
Chlamydomonas	0.26	0.04	6.91	0.29	0.53	141
Chlorella	0.38	0.03	12.8	0.41	0.73	120
Monoraphidium	0.24	0.04	6.15	0.16	0.53	136
Scenedesmus	0.21	0.04	5.34	0.21	0.40	126
Raphidocelis	0.02	0.04	0.52	0.01	0.43	77

S8. Nature of species interactions

We used our experimental data to investigate the nature of the interactions between our species pairs. In its strictest definition, interspecific competition involves any mechanism whereby the fitness (e.g. per-capita rate of increase or population density) of a given species is reduced by the presence of another, for instance because the other species uses more resources. We calculated a relative density (RD) index for each species in each pairwise interaction, according to Fritschie *et al.* (2014). Relative density of each species, i was the ratio of species i's population density in its biculture:monoculture ratio. Ratios below 1 indicate competitive interactions because the density in bi-culture is than than the species was able to acheive when in monoculture – i.e. it incurs a fitness cost due to interspecific competition. Conversely, ratios over 1 indicate facilitation because the focal species achieves a greater density when in the presence of another taxon than it was able to reach when alone. We found that 78.8% of pairs fell into the mutual competition scenario (where $RD_i < 1$), while 17.5 % of pairs fell into an intermediate scenario where one species facilitated while the other species did not, and 3.7 % of pairs fell into a full facilitation scenario (Fig. S8A). Thus, interactions in our experiment are mainly competitive *sensu stricto*. Note that RD is a property of individual species, and any two species grown in biculture may have very different values of RD due to asymmetry in interaction strength.

We also computed another metric of interaction strength, the deviation from expected total yield ΔY, which is a property of the community. To do so, we computed the total cell density of the two species grown in competition and the total cell density of each species grown isolation. We calculated a deviation from expected yield ΔY according to Loreau & Hector (2001), as

$$\Delta Y = Y_o - Y_e = \sum_i RY_{oi} - \sum_i RY_{ei},$$

where Y_o is the observed yield of the two-species mixture at day 14 (in cells·mL^{-1}), Y_e is the expected yield of the two-species mixture, and RY_{oi} and RY_{ei} are the observed and expected relative yields of species i in the mixture. The expected relative yield of species i in the mixture are equal to half of the yield observed in monoculture (as they theoretically have access to half of the nutrients in a two-species mixture). We studied whether the deviation from expected yield varied with species identity (Table S8A). Positive deviations indicate complementarity effects (e.g., niche partitioning or facilitation) while negative deviations indicate competitive interactions diminishing total biomass. In line with the RD calculations the vast majority interactions were negative, indicating strong resource competition characterised the interactions among these 6 species of algae. Interactions involving *Raphidocelis* were strongly negative, while interactions involving *Scenedesmus* were less negative and there was no distinguishable negative interaction for *Chlamydomonas* (Table S8A).

References

Fritschie, K.J., Cardinale, B.J., Alexandrou, M.A. & Oakley, T.H. (2014). Evolutionary history and the strength of species interactions: testing the phylogenetic limiting similarity hypothesis. *Ecology*, 95, 1407–1417.

Loreau, M. & Hector, A. (2001).Partitioning selection and complementarity in biodiversity experiments.*Nature*, 412, 72–76

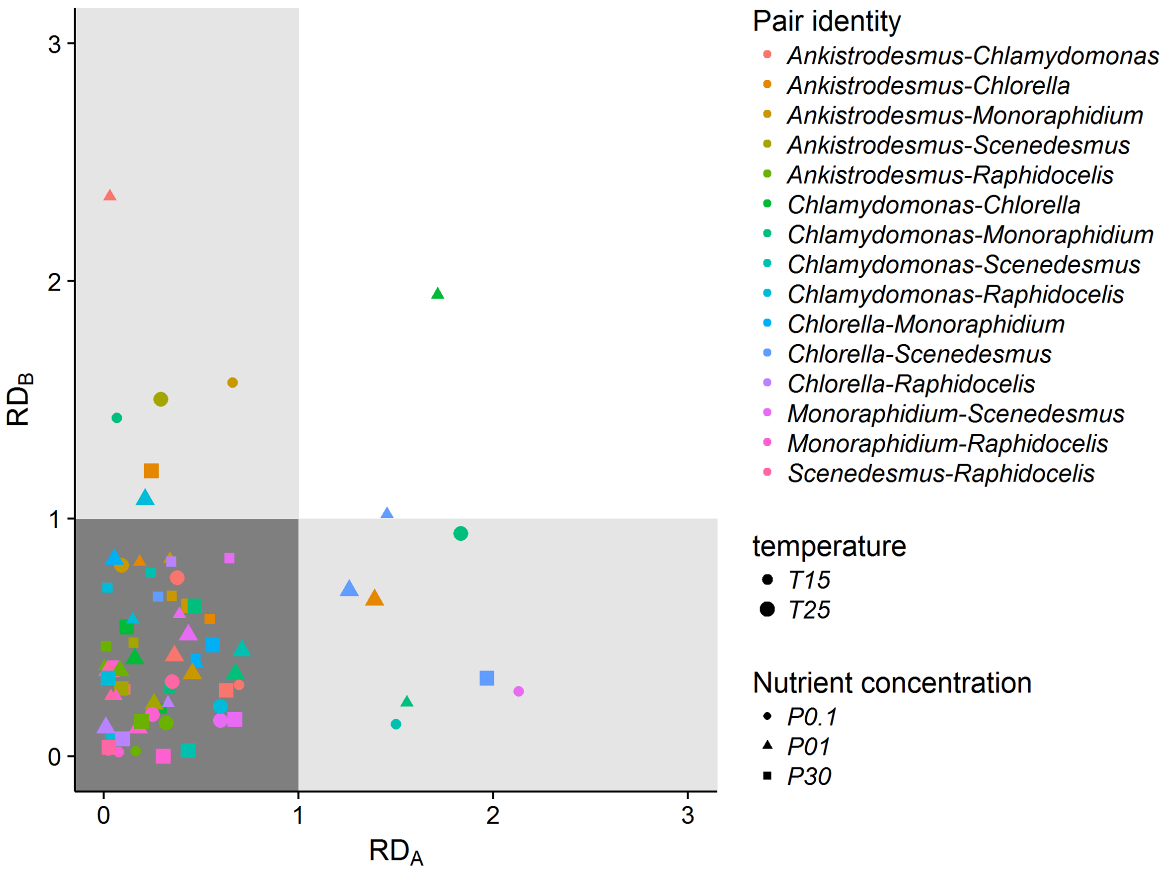

Figure S8A. Distribution of algal communities across an interaction gradient.
Joint distribution of species relative densities (each data point is the mean across replicates of a single biculture at a specific temperature and nutrient level). Relative density (RD) is the mean density of the focal species in competition divided by its mean density when cultivated in isolation. The background colour indicates a gradient of competition strength, dark grey indicates that both species experienced stronger interspecific versus intraspecific competition ($RD_i < 1$, N = 63), while the white background indicates that both species were facilitated ($RD_i > 1$, N = 3). A small subset of interactions fell in interaction scenarios (light grey) where one species was facilitated while the other experienced interspecific competition ($RD_i < 1$, $RD_j > 1$, N = 14). Note that interactions involving *Chlorella* at the very low (P = 0.1) nutrient concentration where removed as the isolates for this species fell below the threshold of 500 cells mL^{-1} (see Methods).

Table S8A. Deviation from the expected yield per species at day 14.
Values from two tailed t-test of $\log_{10}(\Delta Y)$.

**Species**	**mean**	**Confidence interval**	**t-value**	**df**	**pvalue**
Ankistrodesmus	-0.32	[-0.42,-0.23]	-6.99	179	>0.001***
Chlamydomonas	-0.04	[-0.13,0.04]	-1.08	177	0.281
Chlorella	-0.13	[-0.23,-0.04]	-2.76	169	0.006**
Monoraphidium	-0.11	[-0.18,-0.04]	-3.13	174	0.002**
Scenedesmus	-0.10	[-0.19,-0.001]	-2.00	174	0.046*
Raphidocelis	-0.57	[-0.67,-0.47]	-11.5	169	>0.001***

S9. Competitive advantage at day 5 and day 23

In addition to our main results at day 14 of the competition experiment, we also measured competitive advantage at day 5 and day 23. We trained linear discrimination algorithms on isolate data collected at day 5 for day 5 and day 14 for day 23 respectively, except for the very low phosphate concentration for which we trained the discrimination algorithm on the whole dataset to have a better discrimination function given the low density of cells. We chose to train the data using isolate data collected at day 14 for day 23 as the lower noise in the training dataset gave better discrimination results. The discrimination algorithms give 78 % accuracy in discriminating between species at day 5, and 70 % at day 23.

We compared the competitive advantage at day 5 and day 23 to the results of the theory (Table S9A and S9B). Because of a technical problem, we lost results from 63 samples out of 90 from 25ºC and PO_4^{3+} = 1 µmol L^{-1} at day 5. Thus comparisons for this phosphate level for day 5 are to be taken with caution due to low sample size. We note that the results are similar between days, with an overall agreement between theory and experiment of 56 % (R_∞) and 66 % (R) at day 5 and of 63 (R_∞)and 68 % (R) at day 23. Further, measured competitive advantage was correlated across days (Pearson r = 0.67 [0.56, 0.75], t = 10.52, df = 137, p = $2e^{-16}$ and r = 0.53 [0.45, 0.62], t = 11.0, df = 293, p > $2e^{-16}$ respectively for correlation between day 5 and 14 and for correlation between day 14 and 23).This suggests that the competitive advantage at 14 day did indeed carry the signature from the exponential growth phase at day 5 where no species were at carrying capacity, and that this carry-over effect was continued over longer time periods, at a time where all species where at carrying capacity (median time to carrying capacity during the growth rate experiment at 15 and 25°C: 11 and 9 days respectively at very low nutrient concentrations (0.1 µmol·L-1 of phosphate), 10.5 and 7 days at low nutrient concentrations (1 µmol·L-1 of phosphate), and 14.5 and 9 at high nutrient concentrations (30 µmol·L-1 of phosphate)). It is noteworthy that the predictive power of the model is lower at day 23 than at day 14, likely due to the lower accuracy of the discrimination algorithm.

Table S9A. Proportion of competitive advantages correctly predicted by theory at day 5 using the linear discrimination algorithm.
Analogous to Table 1 in the main text, but for day 5.

	R_∞		R		N
Full dataset					
	0.56	(0.137)	0.66	(0.007)	192
By temperature					
$T = 15°C$	0.50	(0.458)	0.64	(0.113)	58
$T = 25°C$	0.59	(0.112)	0.67	(0.025)	134
By nutrient					
[P] = 0.1 μmol·L^{-1}	0.36	(0.820)	0.58	(0.262)	84
[P] = 1 μmol·L^{-1}	0.65	(0.182)	0.65	(0.178)	23
[P] = 30 μmol·L^{-1}	0.74	(0.019)	0.74	(0.016)	85
By species					
Ankistrodesmus	0.65	(0.022)	0.86	(0.000)	74
Chlamydomonas	0.53	(0.230)	0.65	(0.052)	57
Chlorella	0.62	(0.054)	0.75	(0.024)	63
Monoraphidium	0.53	(0.252)	0.71	(0.001)	72
Scenedesmus	0.49	(0.547)	0.57	(0.140)	68
Raphidocelis	0.56	(0.407)	0.32	(0.855)	50

Table S9B. Proportion of competitive advantages correctly predicted by theory at day 23 using the linear discrimination algorithm.

Analogous to Table 1 in the main text, but for day 23.

	R_∞		R		N
Full dataset					
	0.63	(0.000)	0.68	(0.000)	339
By temperature					
$T = 15°C$	0.69	(0.001)	0.66	(0.016)	170
$T = 25°C$	0.57	(0.047)	0.69	(0.001)	169
By nutrient					
[P] = 0.1 $\mu mol \cdot L^{-1}$	0.38	(0.640)	0.92	(0.002)	26
[P] = 1 $\mu mol \cdot L^{-1}$	0.54	(0.251)	0.55	(0.249)	159
[P] = 30 $\mu mol \cdot L^{-1}$	0.77	(0.005)	0.77	(0.005)	154
By species					
Ankistrodesmus	0.63	(0.020)	0.74	(0.000)	123
Chlamydomonas	0.60	(0.022)	0.61	(0.045)	121
Chlorella	0.69	(0.000)	0.73	(0.000)	115
Monoraphidium	0.61	(0.125)	0.66	(0.038)	119
Scenedesmus	0.66	(0.009)	0.66	(0.023)	112
Raphidocelis	0.61	(0.016)	0.67	(0.002)	88
